# Genetic Architecture of Flowering Phenology in Cereals and Opportunities for Crop Improvement

**DOI:** 10.3389/fpls.2016.01906

**Published:** 2016-12-19

**Authors:** Camilla B. Hill, Chengdao Li

**Affiliations:** ^1^Western Barley Genetics Alliance, Western Australian State Agricultural Biotechnology Centre, School of Veterinary and Life Sciences, Murdoch University, PerthWA, Australia; ^2^Department of Agriculture and Food Western Australia, South PerthWA, Australia

**Keywords:** flowering time, phenology, photoperiod, yield, barley, wheat, maize, rice

## Abstract

Cereal crop species including bread wheat (*Triticum aestivum* L.), barley (*Hordeum vulgare* L.), rice (*Oryza sativa* L.), and maize (*Zea mays* L.) provide the bulk of human nutrition and agricultural products for industrial use. These four cereals are central to meet future demands of food supply for an increasing world population under a changing climate. A prerequisite for cereal crop production is the transition from vegetative to reproductive and grain-filling phases starting with flower initiation, a key developmental switch tightly regulated in all flowering plants. Although studies in the dicotyledonous model plant *Arabidopsis thaliana* build the foundations of our current understanding of plant phenology genes and regulation, the availability of genome assemblies with high-confidence sequences for rice, maize, and more recently bread wheat and barley, now allow the identification of phenology-associated gene orthologs in monocots. Together with recent advances in next-generation sequencing technologies, QTL analysis, mutagenesis, complementation analysis, and RNA interference, many phenology genes have been functionally characterized in cereal crops and conserved as well as functionally divergent genes involved in flowering were found. Epigenetic and other molecular regulatory mechanisms that respond to environmental and endogenous triggers create an enormous plasticity in flowering behavior among cereal crops to ensure flowering is only induced under optimal conditions. In this review, we provide a summary of recent discoveries of flowering time regulators with an emphasis on four cereal crop species (bread wheat, barley, rice, and maize), in particular, crop-specific regulatory mechanisms and genes. In addition, pleiotropic effects on agronomically important traits such as grain yield, impact on adaptation to new growing environments and conditions, genetic sequence-based selection and targeted manipulation of phenology genes, as well as crop growth simulation models for predictive crop breeding, are discussed.

## Introduction

An essential foundation of agriculture was the domestication of grasses from the Poaceae family about 15,000 years ago in the Fertile Crescent of the Eastern Mediterranean region (Gill et al., 2004). Since then, grasses have diversified across a range of ecological niches and are now cultivated in each of the different world climates. Barley (*Hordeum vulgare* L.) and rye (*Secale cereale* L.) are adapted to cooler temperate regions, wheat (*Triticum aestivum* L.) and oats (*Avena sativa* L.) to temperate regions, and rice (*Oryza sativa* L.) and maize (*Zea mays* L.) to tropical and warmer temperate zones. Wheat, rice, and maize provide about two-thirds of all energy in human diets, whereas barley grain contributes to the human diet indirectly as animal feed, and directly as a substrate for malting, brewing, and distilling industries (Cassman, 1999). These four cereal crops are central in current efforts to increase arable crop yield and other agronomic outputs in an attempt to secure future food supply security for an increasing world population under a changing climate (Rötter et al., 2015).

Flowering sets the switch from vegetative to reproductive development. The genetic regulation of flowering time is more sensitive to environmental cues than many other agriculturally relevant traits. A crucial phenological developmental step of crops is the floral initiation and timing of flowering, also known as heading or ear emergence date and defined as first anther bust on spikes in cereals. Heading date and photoperiod sensitivity are the fundamental traits that determine adaptation to geographic environments and different cropping systems, and are controlled by an endogenous genetic network as well as environmental cues including day length (photoperiod) and temperature (Andres and Coupland, 2012). The optimal timing of this transition directly affects grain yield as it needs to occur during specific seasons to avoid abiotic (such as cold, frost, heat, and drought) and biotic stresses (including fungi, bacteria, viruses, nematodes, or insects).

Considerable differences exist between cereals grown in temperate and tropical climates, as these climates have different seasons best suited for reproduction. In tropical regions, the majority of plants flower when the days become shorter during the cooler seasons of the year to avoid hot temperatures during summer and are described as short-day (SD) plants. By contrast, among many temperate species, flower development is controlled in response to changes in both photoperiod and prolonged exposure to cold temperature (vernalization). This ensures that autumn-sown crops flower during spring when growth conditions are more favorable than during winter (Greenup et al., 2009). Floral induction is delayed under short day conditions (8 h of light), and enhanced under long day conditions (16 h of light); therefore, many temperate species are described as long-day (LD) plants. Compared to autumn-sown winter cultivars, spring cultivars are sensitive to low temperatures, insensitive to photoperiod, and do not require vernalization (von Zitzewitz et al., 2005). In day-neutral plants, such as temperate maize (*Zea mays* ssp *mays*) cultivars, there is no specific photoperiod requirement as flowering is promoted almost exclusively through the coordinated action of autonomous regulatory pathway genes (Mascheretti et al., 2015).

The topic of this review is to summarize, compare and contrast the current understanding of flowering time regulation in the four major cereal crop species wheat, barley, rice, and maize. We will highlight how different flowering time regulatory mechanisms have evolved between SD, LD, and day-neutral plants and how evidence is accumulating that miRNA and epigenetic regulation play a major role in controlling phenology gene action in cereals. We will describe how genetic variation in flowering time genes paved the way for adaptation to new growing environments. A detailed understanding of flowering mechanisms is the cornerstone of future developments for genetic sequence-based selection and targeted manipulation of phenology genes. In the final part of this review, we will discuss how this knowledge can inform crop simulation models as well as gene-based models developed to predict phenology, the current status of development and use of these models, and how their findings can translate into an improved yield on the field.

## A Brief History of Flowering Time

Like the cereal crops wheat and barley, *Arabidopsis* is an LD plant sensitive to vernalization and photoperiod and adapted to temperate climates (Greenup et al., 2009). With some marked exceptions, genes and proteins with analogous function and similar molecular mechanisms found in *Arabidopsis* also control seasonal flowering responses in temperate grasses. These genes and proteins are part of complex signaling pathways that revolve around a set of main flowering time integrator genes that react to endogenous triggers including genes belonging to the circadian clock, autonomous, age, and gibberellin pathways, as well as environmental signals such as ambient temperature and photoperiod. Since recent reviews give comprehensive descriptions of flowering genes and mechanisms in *Arabidopsis*, the model plant for flowering time control (Song J. et al., 2013; Ó’Maoiléidigh et al., 2014; Johansson and Staiger, 2015; Song et al., 2015), only a brief overview of the main integrator genes is given in this section. This will provide a basis for a more detailed discussion of gene networks controlling cereal phenology in bread wheat, barley, rice, and maize, particularly for components absent in *Arabidopsis*.

### FLOWERING LOCUS T: Uncovering the Identity of Florigen

For nearly 80 years, researchers were trying to characterize the elusive florigen, a hormone-like substance believed to be responsible for promotion or stimulation of flowering in plants. The term was first coined by Russian scientist Chailakhyan (1936) while experimenting on phototropism. Early grafting experiments demonstrated that this floral signal is produced in the leaves, and transferred to the shoot apical meristem (SAM) through the phloem (Zeevaart, 1976). However, after these initial successes, the exact identity of florigen remained unknown until the beginning of the 21st century when the first plant genome sequences were made publicly available and provided the genetic tools to investigate flowering time in more detail. In 2005, three independent groups from Germany (Wigge et al., 2005), Japan (Abe et al., 2005), and Sweden (Huang et al., 2005) reported having identified a gene that controls flowering time named *FLOWERING LOCUS T* (*AtFT*). Published in the journal *Science*, Huang et al. (2005) reported *AtFT* mRNA as the transmissible signal required for flowering. However, the journal article was retracted after 2 years (Bohlenius et al., 2007) as the results could neither be supported by other research groups (Lifschitz et al., 2006) nor could be replicated in their laboratory (Huang et al., 2005). Many more genetic components of flowering time were identified in the following years (Conti and Bradley, 2007; Sawa et al., 2007), but none provided evidence of how FT is transported from leaves to the shoot apex. In 2007, studies in *Arabidopsis* (Corbesier et al., 2007) and rice (Tamaki et al., 2007) independently reported that the FT protein itself is the mobile flowering signal, supporting the previous results found in tomato (Lifschitz et al., 2006).

**FIGURE 1:**
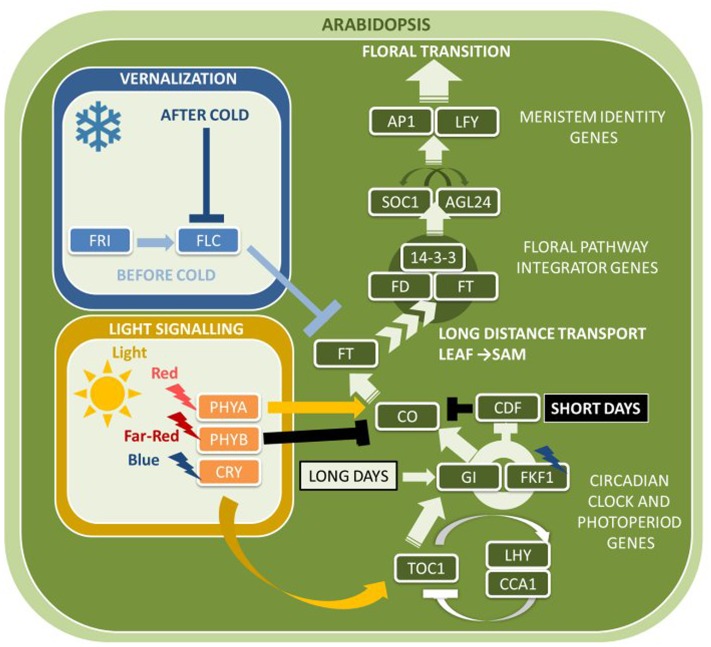
**Major flowering pathway genes of *Arabidopsis thaliana*.** Positive and negative regulatory connections are indicated by arrows and lines with T-ends, respectively. White and black arrows or T-ends indicate regulatory connections occurring primarily under long days and short days, respectively. Gene name abbreviations are explained in **Supplementary Table 1**.

According to the current status of research in *Arabidopsis* and rice, FT protein travels via the phloem to the SAM, where it binds to bZIP transcription factors FLOWERING LOCUS D (AtFD) and FD PARALOG (FDP) (Abe et al., 2005; Wigge et al., 2005) (**Figure 1**). This activates the expression of several floral meristem identity genes that induce the transition toward reproductive development. A central floral meristem identity gene is *LEAFY* (*AtLFY*), which is activated by transcription factors *AGAMOUS-LIKE24* (*AtAGL24*), *SUPPRESSOR OF OVEREXPRESSION OF CONSTANS1* (*AtSOC1*), and *SHORT VEGETATIVE PHASE* (*AtSVP*). AGL24 and SVP were shown to enhance expression of a MADS-box transcription factor encoding gene *APETALA 1* (*AtAP1*), as well as two genes closely related to *AP1*, *CAULIFLOWER* (*AtCAL*) and *FRUITFUL* (*AtFUL*) (Ferrandiz et al., 2000; Wigge et al., 2005; Grandi et al., 2012; Jaeger et al., 2013). In addition to activating the expression of floral meristem identity genes, *AtLFY* and *AtAP1* also repress negative regulators of *AtFT*, such as *TERMINAL FLOWER1* (*AtTFL1*), *TEMPRANILLO1* (*AtTEM1*) and *AtTEM2* (Kaufmann et al., 2010).

### Gibberellin: A Key Phytohormone Influencing Flowering Time

Gibberellins (GAs) are diterpene phytohormones that regulate multiple aspects of plant growth, including flower and seed development (Yamaguchi, 2008). Many GA metabolism enzymes are multifunctional, accepting several substrates to produce a diverse set of GAs. Only a subset of all GAs has a bioactive function, including GA_1_, GA_3_, and GA_4_. Different forms of GA are converted via the action of a series of various enzymes, most notably encoded by *AtGA20OX* genes which catalyze the conversion of non-bioactive to bioactive forms of GA. In *Arabidopsis*, GA signaling is required at the apex during floral induction and involves binding of GA to the GIBBERELLIN INSENSITIVE DWARF1 (AtGID1) receptors to promote degradation of DELLA proteins via the ubiquitin proteasome pathway (Murase et al., 2008). GA further influences flowering time by regulating the expression of *AtSOC1* and *AtLFY* (Moon et al., 2003). Very recently, *SQUAMOSA PROMOTER BINDING PROTEIN-LIKE 15 (AtSPL)* was demonstrated to be regulated by miR156 and to promote flowering under non-inductive conditions (Hyun et al., 2016).

### CONSTANS: Operator of the Flowering Time Checkpoint

Genetic regulatory mechanisms of flowering time consist of a series of feedback loops through which individual components of the circadian clock sequentially or reciprocally repress each other (Johansson and Staiger, 2015). Putterill et al. (1995) identified a late-flowering mutant under LD conditions and cloned the corresponding *CONSTANS* (*AtCO*) gene. The corresponding protein CO is a nuclear B-Box zinc-finger (BBX) protein with a C-terminal CCT (CO, CO-like, and TIMING OF CAB EXPRESSION 1, TOC1) DNA binding domain, located in the leaves, and responsible for regulating flowering in response to day length.

In addition to genetic regulation via feedback circuits, post-translational processes contribute to adjusting clock protein oscillations to LDs. Under SDs, *CO* expression is repressed in the morning by CYCLING DOF FACTOR (AtCDF) proteins that bind to its promoter (Fornara et al., 2009). During the day, *AtCO* mRNA levels increase with a peak before dusk but are ultimately degraded at night so that *AtFT* expression remains repressed. In SDs, GIGANTEA (AtGI) and FLAVIN BINDING, KELCH REPEAT, F-BOX1 (AtFKF1) clock proteins are asynchronously expressed and do not interact, but synchronize their expression under LDs (Sawa et al., 2007). AtFKF1 recognizes AtCDF proteins via its Kelch-repeat domain, whereas the LOV domain absorbs blue light, crucial for the interaction with AtGI. Only in response to LDs, AtCDF degrades via the ubiquitin-proteasome pathway through the action of the GI-FKF1 complex. As a result, *AtCO* mRNA can accumulate during the day to induce transcription of *AtFT*, and the AtFT protein is then transferred via the phloem to the SAM (Corbesier et al., 2007).

### CRYPTOCHROMES and PHYTOCHROMES: Timekeepers of the Endogenous Clock

AtCO is regulated at the protein-level by phytochromes and cryptochromes that either stabilize or destabilize AtCO mRNA (Devlin, 2002). The blue-light-dependent interaction of AtFKF1with AtGI to antagonistically control *CO* transcript stability is one example of a plant molecular mechanism that adjusts the period of clock protein oscillations to a 24-h rhythm. Depending on the intensity, periodicity, and spectral quality (such as blue, red and far-red light) of incoming sunlight, photosensory pigments trigger transduction chains that alter gene activity of clock genes further downstream (Fankhauser and Staiger, 2002). In addition to AtFKFI, its homologs ZEITLUPE (AtZTL) and LOV KELCH PROTEIN 2 (AtLKP2), as well as the red/far-red-absorbing PHYTOCHROMES A–E (AtPHYA–PHYE) and the blue light-absorbing CRYPTOCHROMES (AtCRY1, AtCRY2) modulate gene activity to influence plant growth and development (Devlin, 2002).

Changes in light and temperature at dawn and dusk reset the circadian clock, thus allowing the plant to adjust physiologically and metabolically to changing day lengths during different seasons of the year, crucial for ensuring flowering occurs at the optimal time. Very recently, a molecular mechanism was proposed by which endogenous circadian clocks converge with light-signaling pathways through the interaction of AtTOC1 and PHYTOCHROME-INTERACTING FACTORS (AtPIFs), which belong to a subfamily of basic helix–loop–helix transcription factors (Soy et al., 2016). *Arabidopsis* phytochromes AtPHYA–PHYE regulate the PIF pathway and downstream targets of PIF by inducing degradation of AtPIF1, AtPIF3, AtPIF4, and AtPIF5 (Leivar and Monte, 2014). While the phytochromes remain photo-activated during the day, PIF levels remain low, but increase when the levels of photoactivated phytochromes decline particularly during long nights (Soy et al., 2012). AtPIF protein level oscillations facilitate timing of hypocotyl elongation growth rates to the optimum time just before dawn when AtPIF is most abundant in the circadian cycle. *AtTOC1* directly represses the transcriptional activator activity of AtPIF3 protein in SDs after dusk by binding to the *AtPIF3* promoter despite high AtPIF3 abundance (Soy et al., 2016). This mechanism regulates dawn-phased growth-related and hormone-associated genes in controlling early hypocotyl growth in diurnal SD conditions.

### FLOWERING LOCUS C: Major Flowering Repressor with an Epigenetic Memory for Cold

The major repressor of flowering is the MADS-box transcription factor gene *FLOWERING LOCUS C* (*AtFLC*) (Song Y.H. et al., 2013). *AtFLC* is expressed in mitotically active regions such as the SAM and the root apical meristem, the primary tissues of cold perception. When active alleles of the flowering time regulator gene *FRIGIDA* (*AtFRI*) are present, *AtFLC* expression is enhanced in a dose-dependent manner up to a level that prevents flowering (Michaels and Amasino, 1999). The promotion of flowering after exposure to prolonged cold temperatures, a process termed vernalization which occurs during winter or can be induced artificially, silences AtFLC and allows flowering in the following spring. Such silencing was shown to involve a Polycomb-based epigenetic memory system and requires polycomb repressive complex 2 (AtPRC2)-mediated methylation of H3K27 to form facultative heterochromatin, which allows for the maintained AtFLC repression even after warmer conditions have returned (Bastow et al., 2004; Wood et al., 2006). The extensive natural variation present for both the vernalization requirement and response to cold has played a key role in the adaptation of many plant species over a wide latitudinal range. For example, naturally occurring *AtFLC*, *AtFRI, and AtPHYC* polymorphisms result in varying flowering times and are involved in adaptations to different climates in *Arabidopsis* (Mendez-Vigo et al., 2011).

## Flowering Time in Cereal Crops: A Complex Interplay Between Genes and Environment

Although studies in the model dicot *Arabidopsis* were fundamental to deciphering flowering time mechanisms, recent research in rice has extended our knowledge of flowering processes to a model monocot species. However, considerable differences between cereals grown in temperate and tropical climates exist, thus compromising the relevance of rice as a model cereal for phenology. As an example, key regulatory genes for long day and short day photoperiodic control of flowering are conserved in the SD plant rice and LD plants wheat and barley, but the regulation of these genes is often reversed (Hayama et al., 2003).

The release of genome sequences for rice (Goff et al., 2002), maize (Schnable et al., 2009), barley (International Barley Genome Sequencing Consortium, 2012), and bread wheat (Mayer et al., 2014), coupled with advancements in molecular biology techniques [including DNA sequencing, RNA interference, and quantitative trait locus (QTL) analysis] as well as comparative genetics approaches, have enabled the isolation and functionally characterization of many phenology genes in cereal crops. Blast searches and phylogenetic analyses between *Arabidopsis*, rice, wheat, barley, and maize sequences identified many conserved as well as functionally divergent genes involved in flowering (Calixto et al., 2015). Several key pathways linked to specific environmental signals were uncovered and characterized, including temperature (thermoperiod and vernalization), day length (photoperiod), as well as autonomous and gibberellin-responsive pathways. More recently, miRNA-based regulation of gene expression at the post-transcriptional level has come into the focus of research efforts on flowering-related pathways (Teotia and Tang, 2015). In this section, we will summarize phenology genes and genetic regulation, and highlight how different flowering time regulatory mechanisms have evolved between SD, LD, and day-neutral plants.

### Regulation of Flowering Time in Long-Day Plants Bread Wheat and Barley

Since originating from the Eastern Mediterranean region, the closely related Triticeae cereal crops bread wheat and barley have been adapted to a wide range of agricultural environments. Traditional breeding and phenotypic selection of natural genetic variants at flowering loci was used to optimize flowering time within a given production environment to achieve greater yields (Jung and Müller, 2009). This selection was also often associated with improved adaptations to abiotic and biotic stress factors.

**FIGURE 2:**
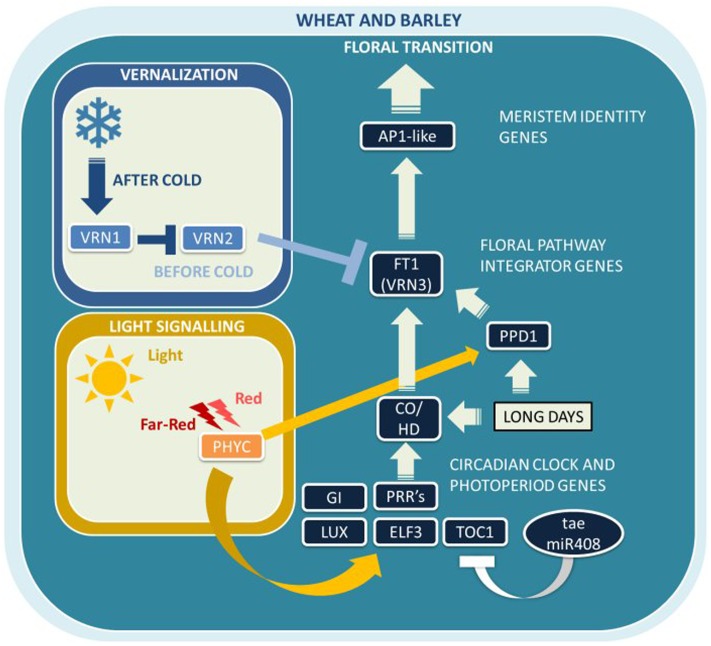
**Major flowering pathway genes of bread wheat (*Triticum aestivum L.*) and barley (*Hordeum vulgare* L.).** Positive and negative regulatory connections are indicated by arrows and lines with T-ends, respectively. White arrows or T-ends indicate regulatory connections occurring primarily under long days. Gene name abbreviations are explained in **Supplementary Table 1**.

Bread wheat and barley are facultative LD plants adapted to short growing seasons, with LDs promoting flowering in spring, while SDs delay reproductive development. Photoperiod and vernalization regulate the initiation of the reproductive phase and are the two main seasonal signals in temperate cereal crops. Many Pooid grasses, including wheat and barley, are vernalization responsive and photoperiod sensitive, which is believed to have evolved early during diversification as a crucial adaptation to allow for their transition into the temperate zone (Fjellheim et al., 2014). Temperate regions with long growing seasons and sufficient water supply allow cereal crops to flower late in the year and accumulate more biomass, whereas early flowering avoids abiotic and biotic stresses such as drought and pathogen attack late in the season. To date, only a relatively small number of major flowering time genes and pathways have been identified in wheat and barley (**Figure 2**). The following two sections on wheat (Flowering Time in Bread Wheat) and barley (Flowering Time in Barley) will give a detailed account of the current understanding of flowering time regulation in these two temperate cereal crops.

#### Flowering Time in Bread Wheat

##### Genetic regulation of photoperiod response

The process of wheat domestication involved several hybridization events between tetraploid progenitor species *Triticum turgidum* L. (AABB genomes) and diploid *Aegilops tauschii* (DD genome) that gave rise to hexaploid bread wheat (AABBDD genomes) about 8,000 years ago (Wang et al., 2013). As a hexaploid, bread wheat displays a high level of gene redundancy (one copy of the homoeologous gene in each genome), and therefore the precise function of many flowering-related genes and the current understanding of the complexity of the gene networks controlling flowering is still incomplete. **Table 1** provides a summary of the current understanding of wheat phenology genes which are either functional orthologs or homologs of *Arabidopsis thaliana* genes.

**Table 1:** Wheat functional orthologs or homologs of *Arabidopsis thaliana* genes.

Wheat	*Arabidopsis*	Function	Chromosome	Reference
**Functional orthologs**				
*TaCO1/TaHD1*	*AtCO*	Flowering promoter under inductive LD conditions	4A	Nemoto et al., 2003; Shimada et al., 2009
*TaFT1 (VRN3)*	*AtFT*	Flowering promoter	5D	Yan et al., 2006
*TaGI1*	*AtGI*	Flowering promoter	3H	Zhao et al., 2005
*TaRHT-B1*, *TaRHT-D1*	*AtGAI*	Gibberellin metabolism	2D	Pearce et al., 2013; Boden et al., 2014
**Homologs with pleiotropic or divergent function**				
*TaTOC1*	*AtTOC1/AtPRR1*	tae-miR408-mediated oscillation regulator, affects flag leaf angle and plant height	6A/6B/6D	Zhao et al., 2016
*TaPPD1*	*AtPRR7*	Photoperiod sensitivity and flowering time	2D	Beales et al., 2007
*TaVRN1*	*AtAP1/AtCAL/AtFUL*	Flowering promoter in response to vernalization	5A	Yan et al., 2003; Shimada et al., 2009
*TaVRN2*	*AtCOL*	Flowering repressor	4B, 5A	Yan et al., 2004
*TaVRN4*	*AtAP1/AtCAL/AtFUL*	Paralog of *TaVRN-A1*, modulates vernalization response	5D	Kippes et al., 2015


*Functional orthologs have a conserved function; homologs have a pleiotropic or divergent function. Gene name abbreviations are explained in **Supplementary Table 1**.*

Rapid genomic changes in polyploid wheat facilitated wheat adaptation to different growing environments. In particular, wheat crop expansion is associated with the exploitation of natural variation in the photoperiod (*PPD*), vernalization (*VRN*), and *EARLINESS PER SE* (*EPS*) genes. In bread wheat, *PPD* genes are relatively well understood and molecular markers have been developed to aid selection for breeders (Beales et al., 2007; Cane et al., 2013). Three copies of the gene (homeologs) that control photoperiod response in wheat, namely *TaPPD-A1*, *TaPPD-B1* and *TaPPD-D1*, are located on the short arm of the homoeologous group 2 chromosomes. The region containing *TaPPD-D1* in wheat is colinear with the barley photoperiod gene *HvPPD-H1* on chromosome 2HS (Laurie, 1997). *TaPPD-D1* encodes a pseudo-response regulator (PRR) family protein gene orthologous to the *Arabidopsis PRR*7 gene, and is more distantly related to *AtCO* and *AtVRN2* (Yan et al., 2004; Turner et al., 2005).

The wild ancestors of wheat were photoperiod-sensitive, and photoperiod insensitivity in many cultivated wheat varieties is the result of mutations in *PPD* genes (Thomas and Vince-Prue, 1997). Dominant *PPD* alleles induce constitutive activation of the photoperiod pathway irrespective of day length and significantly reduce sensitivity to photoperiod. This leads to an early flowering phenotype in both SDs and LDs, which has been associated with pleiotropic effects in certain agricultural environments including in southern Europe (Worland, 1996) and Australia (Richards et al., 2014) resulting in increased grain yields. The potencies of the homoeologous group-2 *PPD* genes for insensitivity are ranked in the order *TaPPD-D1 > TaPPD-B1 > TaPPD-A1* (Worland et al., 1998).

Pseudo-response regulator proteins are commonly found in plants and involved in circadian clock-associated pathways, including PRR1 (TOC1) (Mizuno and Nakamichi, 2005). The mRNA of *TOC1* starts to accumulate at the beginning and reaching its maximum level at the end of the light period, where it directly represses the transcription of the myeloblastosis (MYB) transcription factors *LATE ELONGATED HYPOCOTYL* (*LHY*) and *CCA1*. In wheat, Zhao et al. (2016) recently demonstrated that the microRNA tae-miR408 targets and down-regulates expression levels of all three wheat paralogs of *TaTOC1 (TaTOC-A1*, *TaTOC-B1*, and *TaTOC-D1)* under both LDs and SDs, thereby regulating heading time and associated agronomic traits.

##### Genetic regulation of vernalization response

The extensive natural variation for both the requirement and responsiveness to cold temperature has played a major role in the adaptation of many species over a wide latitudinal range. Unlike the model monocot crop rice, wheat and barley flower in response to LDs. The photoperiod and vernalization pathways intersect at the regulation of *TaVRN3*, which is a gene encoding a phosphatidylethanolamine-binding protein (PEBP) homologous to the *Arabidopsis* flowering time gene *AtFT1* (Yan et al., 2006). Similarly, *VRN3* also encodes a mobile protein that is transported from the leaves to the SAM, where it becomes part of a flowering complex that induces *TaVRN1* transcription by binding to its promoter. *TaVRN1* is an AP1 clade MADS-box transcription factor, homologous to the *Arabidopsis* meristem identity gene *AtAP1, and* present on the long arms of chromosomes 5A, 5B, and 5D, respectively (*TaVRN-A1*, *TaVRN-B1*, and *TaVRN-D1)* (Yan et al., 2003). While vernalization in *Arabidopsis* results in silencing of the flowering repressor AtFLC, it induces transcription of the flowering activator *TaVRN1* in wheat and other monocots. *TaVRN1* is expressed in the leaves where it represses transcription of *TaVRN2*, which encodes a protein with a putative zinc finger and a CCT protein–protein interaction domain (Yan et al., 2004; Kippes et al., 2015). *TaVRN2* represses *TaVRN3 expression in LDs* to prevent flowering during unfavorable conditions in autumn before vernalization. In the absence of *TaVRN2*, the increase in day length during spring results in the up-regulation of *TaVRN3* expression, which leads to a further increase of *TaVRN1* transcription trough a positive feedback loop, and ultimately to an irreversible acceleration of flowering.

A fourth vernalization locus, *TaVRN-D4*, was recently identified on the short arm of chromosome 5D (Kippes et al., 2015). It was shown to be paralogous to *TaVRN-A1*, as it originated from an insertion of a large segment on chromosome 5AL containing the *TaVRN-A1* gene into chromosome 5DS. The first intron of *TaVRN-A1* contains binding sites for the RNA-binding protein *GLYCINE-RICH RNA-BINDING PROTEIN* 2 (TaGRP2), which binds to *TaVRN-A1* pre-mRNA and represses *TaVRN-A1* expression (Xiao et al., 2014). The inserted copy of *TaVRN-A1* carries mutations in its coding and regulatory regions including single nucleotide polymorphisms (SNPs) in the first intron that impedes binding of *TaGRP2*. Protein modification of TaGRP2 by *O*-linked β-*N*-acetyl glucosamine (*O*-GlcNAcylation) mediates interaction with VERNALIZATION-RELATED 2 (TaVER2), a carbohydrate-binding jacalin lectin, which relieves repression of *TaVRN1* expression via TaGRP2 when vernalization occurs.

The genetic regulation of vernalization response in wheat (and other monocots) has some distinct differences from *Arabidopsis*. Comparative genomics studies detected *Arabidopsis FLC*-like genes in monocots, but they have yet to be shown to produce proteins of similar function in wheat or other temperate cereals (Ruelens et al., 2013). *AtFLC* is positively regulated by *AtFRI*, but to date also no functional *AtFRI* homologs were detected in wheat. The *AtFT1* and *AtFRI* promoters and first introns were shown to contain *cis*-regulatory sites important for the transcriptional regulation of these genes in *Arabidopsis* (Tiwari et al., 2010; Sanchez-Bermejo and Balasubramanian, 2015).

Only comparatively little is currently known about genetic sequence variations and non-gene elements that regulate phenology gene expression in wheat. Autumn-sown winter cultivars that contain the ancestral *VRN1* allele require vernalization during winter. By contrast, spring wheat varieties carrying mutations in regulatory regions including in the *TaVRN1* promoter, first intron of *TaVRN1*, or in the *TaVRN4* region (Fu et al., 2005; Kippes et al., 2015; Muterko et al., 2015), do not require vernalization and are sown in spring. Very recently, Kippes et al. (2016) developed a triple *TaVRN2* mutant that contained three non-functional *TaVRN2* alleles. This mutant flowered early, had a limited vernalization response, and spring growth habit. In a different study, an insertion in the promoter of *TaVRN3* led to increased *TaVRN3*, bypassing the delay of flowering by *TaVRN2* (Yan et al., 2006). Sequence variations in *TaVRN3* genes were shown to cause early flowering in wheat (Ó’Maoiléidigh et al., 2014). As another recent example, copy number variation (CNV) was shown to play a major role in global wheat adaptation. Wheat lines with an increased copy number of *TaVRN-A1 showed* an increased requirement for vernalization, and lines with an increased copy number of *TaPPD-B1* led to early flowering (Würschum et al., 2015).

##### Genetic regulation of autonomous pathways

The third class of genes responsible for fine-tuning of wheat flowering time are *EPS* genes, which can be defined as the least number of days to reproductive growth independent of vernalization and photoperiod. Also known as earliness in the narrow sense, intrinsic earliness, or basic development rate, *EPS* genes are hypothesized to function as a fine tune adjustment of flowering time (Zikhali and Griffiths, 2015). They are often considered as polygenic and of small genetic effect, and can be detected in both winter and spring wheats. Variation in *EPS* genes was shown to lead to minor or major differences in flowering time under field conditions, reported in the range of a few days up to a few weeks (Appendino and Slafer, 2003; Zikhali et al., 2014). To date, no *EPS* genes have been cloned, there is only a limited understanding of gene function, and furthermore, no known homologs in *Arabidopsis* exist that could help to fill the knowledge gaps. Instead, several studies used near isogenic lines (NILs) to narrow down the location of *EPS* loci on the wheat genome, and to identify several potential candidate genes (Zikhali et al., 2014, 2015). The genes *MOLYBDENUM TRANSPORTER 1* (*TaMOT1*), *FILAMENTATION TEMPERATURE SENSITIVE H* (*TaFtsH4*) (Faricelli et al., 2010), and *EARLY FLOWERING 3* (*TaELF3*) (Zikhali et al., 2014) were proposed as candidate genes underlying the *EPS -Am1* locus. Gawroński et al. (2014) suggested a cereal ortholog of *Arabidopsis circadian clock gene LUX ARRHYTHMO*/*PHYTOCLOCK 1* (*AtLUX*/*PCL1*) as the candidate gene underlying the *EPS -3A^m^* locus in einkorn wheat (*Triticum monococcum* L.). These recent findings question the previous assumption that *EPS* acts independently of the environment, but the exact nature of the environmental impact on *EPS* gene function remains unclear.

##### Genetic regulation of gibberellin response pathways

Previously, Dubcovsky et al. (2006) showed that an inductive LD photoperiod is needed to induce additional genes for normal spike development as the induction of *TaVRN1* alone proved insufficient. Pearce et al. (2013) hypothesized that GAs may be involved in wheat spike development during LDs, which points to a functionally different role compared with *Arabidopsis* where GAs function in non-inductive SD conditions (Hyun et al., 2016). Pearce et al. (2013) applied exogenous GA to in wheat lines grown under non-inductive SDs, which resulted in an accelerated spike development only wheat lines expressing *TaVRN1*. Both GA and *TaVRN1* are required for the up-regulation of the floral meristem identity genes *TaSOC1* and *TaLFY* and the development of the wheat spike. Furthermore, GA biosynthetic gene expression was found to be elevated in the apices of plants transferred from SDs to LDs as well as in photoperiod-insensitive and transgenic wheat plants with increased *TaFT* transcription under SDs. Wheat genes for components of the GA biosynthetic pathway were identified based on homology with *Arabidopsis*, rice, and *Brachypodium distachyon* (Pearce et al., 2015), but due to the lack of a fully sequenced wheat genome, it is currently not possible to accurately determine the final number of genes in each family.

GA-sensitive and insensitive dwarfing genes have had an impact on all the main cereal crop species (Jia et al., 2009). Semi-dwarfing wheat varieties containing alleles of the *REDUCED HEIGHT* (*RHT)* DELLA genes were key factors to increasing yield during the “Green Revolution” (Flintham et al., 1997; Peng et al., 1999). Reduced crop height protected against lodging and improved harvest index, high spikelet fertility, and grain numbers per ear. *RHT* encodes a negative regulator of the GA signaling pathway (Pearce et al., 2013; Boden et al., 2014).

#### Flowering Time in Barley

##### Genetic regulation of photoperiod response

It is estimated that the common ancestor of barley and *Arabidopsis* shared about two-thirds of the circadian clock and clock-associated genes (Calixto et al., 2015) (**Table 2**). After separation into monocot and dicot classes, these genes further diversified due to extensive gene duplication and deletion events (Campoli et al., 2012a). Although some core clock genes were identified in barley based on map-based cloning (Yan et al., 2003), positional cloning (Yan et al., 2004), or homology with *Arabidopsis* (Yan et al., 2006), only a few are well characterized. Some of these genes were found to perform similar functions as their orthologs, whereas there is currently little evidence for other genes that would support their involvement in flowering.

**Table 2:** Barley functional orthologs or homologs of *Arabidopsis thaliana* genes.

Barley	*Arabidopsis*	Function	Chromosome	Reference
**Functional orthologs**				
*HvCO1*	*AtCO*	Floral promoter	7H	Campoli et al., 2012a
*HvELF3 (EAM8)*	*AtELF3*	Photoperiod sensitivity	1H	Faure et al., 2012; Zakhrabekova et al., 2012
*HvFT1 (VRN3)*	*AtFT*	Floral promoter	7H	Faure et al., 2007
*HvLUX1 (EAM10)*	*AtLUX ARRHYTHMO*	Circadian clock and photoperiodic response	3H	Campoli et al., 2013
*HvPHYC (EAM5)*	*AtPHYC*	Light signaling, photoperiodic regulation	5H	Pankin et al., 2014
**Homologs with pleiotropic or divergent function**				
*HvCEN* (*EPS2* locus)	*AtTFL1*	Flowering time variation, affects yield and thousand kernel weight	2H	Comadran et al., 2012
*HvPPD-H1*	*AtPRR7*	Photoperiod sensitivity and flowering time	2H	Turner et al., 2005; Campoli et al., 2012b
*HvPPD-H2 (HvFT3)*	*AtFT*	Floral promoter under SD conditions, affects grain yield	1H	Cuesta-Marcos et al., 2009
*HvVRN1*	*AtAP1/AtCAL/AtFUL*	Floral promoter in response to vernalization, affects growth rate, spike length, yield	5H	Trevaskis et al., 2006; Rollins et al., 2013
*HvVRN-H2*	*AtCOL*	Floral repressor, affects growth rate, spike length, yield	4H	Trevaskis et al., 2006


*Functional orthologs have a conserved function; homologs have a pleiotropic or divergent function. Gene name abbreviations are explained in **Supplementary Table 1**.*

The major genes controlling flowering time in barley in response to environmental cues are *HvVRN-H1*, *HvVRN-H2*, *HvVRN-H3 (HvFT1)*, *HvPPD-H1*, and *HvPPD-H2 (HvFT3)* (**Figure 2**). The epistatic relationship of three genes involved in flowering regulation is well established: *HvVRN-H1* and *HvVRN-H2*, with roles in vernalization, as well as the floral pathway integrator gene *VRN-H3*, which is synonymous to *HvFT1* (Kikuchi et al., 2009).

Similarly as for *Arabidopsis* and wheat, *FT-like* genes in barley such as *HvFT1* trigger flowering in response to LDs. *HvFT1* plays a key role in promoting flowering and integrates the photoperiod and vernalization pathways: Under LDs, *HvFT1* expression is low until induced by low temperatures in winter varieties that have a vernalization requirement (Yan et al., 2006). *HvFT1* expression is primarily regulated by the major photoperiod response genes *HvPPD-H1* and *HvPPD -H2* (Laurie et al., 1994). Photoperiodic flowering under LDs is up-regulated by *HvPPD-H1*, which is a homolog of the *Arabidopsis* clock gene *PRR7* (Turner et al., 2005). A mutation in the CCT domain of *HvPPD-H1* is associated with lower transcript levels of *HvFT1* and delayed flowering under LDs compared with the wild-type *HvPPD-H1* allele, but it is not related to flowering variation under SDs (Turner et al., 2005; Hemming et al., 2008). *HvPPD-H2* (with its proposed candidate gene *HvFT3*) enhances the expression of *HvFT1* and promotes flowering under non-inductive SDs, whereas recessive mutant alleles confer delayed flowering under SDs, aiding the flowering repression over winter (Kikuchi et al., 2009).

In contrast to *Arabidopsis*, *HvFT1* expression is induced via *HvCO1* (the closest ortholog of *Arabidopsis CO* in barley) in both SDs and LDs (Campoli et al., 2012a). There is a high level of redundancy in both *CO-like* and *FT-like* genes, with currently nine and five members in barley, respectively (Faure et al., 2007). *HvCO1* and *HvCO2* are believed to be paralogs that exist due to a duplication event in temperate cereals. This functional diversification is important for the modulating of flowering responses and adaptation to different growing environments. Several studies demonstrated that functional polymorphisms in the *HvFT1* gene alter its regulation, including mutations in the first intron that differentiate dominant from recessive alleles (Yan et al., 2006), and polymorphisms in the *HvFT1* promoter region that lead to distinct phenotypic effects (Casas et al., 2011).

##### Genetic regulation of vernalization response

In barley, the *VRN-H2* locus consists of three homologous *CO-like* genes, *HvZCCT-Ha*, *HvZCCT-Hb*, and *HvZCCT-Hc* (Dubcovsky et al., 2005; Karsai et al., 2005). *VRN-H2* functions as a floral repressor of *HvFT1* (*VRN-H3*) to delay flowering in plants that have not been vernalized (Trevaskis et al., 2006; Hemming et al., 2008). *VRN-H2* is only expressed under LD conditions controlled by components of the circadian clock. Mutations in *HvELF3* resulted in the expression of *VRN-H2* under SD conditions due to an increased expression of *PPD-H1* and, consequently, *HvFT1* (Faure et al., 2012; Turner et al., 2013).

In winter barley cultivars, vernalization induces *VRN-H1* to repress *VRN-H2* which promotes the transition from vegetative to reproductive development (Trevaskis et al., 2006; Hemming et al., 2008). Sequence variations in the first intron of *VRN-H1* alter the vernalization requirement for activation and, consequently, the repression of *VRN-H2* and overall flowering behavior (Hemming et al., 2008, 2009). A recent survey showed that the down-regulation of *VRN2* by cold is exclusively found in Pooid grasses including wheat and barley (Woods et al., 2016).

By contrast, spring barley cultivars are characterized by natural mutations at PPD-H1 and by deletions of the VRN-H2 locus and thus do not require vernalization (Dubcovsky et al., 2005). HvCO2 overexpression in spring barley was shown to induce flowering due to an increased expression of PPD-H1 and, as a direct result, HvFT1 (Mulki and von Korff, 2016). By contrast, overexpression of HvCO1/CO2 increased HvVRN-H2 expression in winter barley carrying the VRN-H2 locus, which led to lower HvFT1 levels and delayed flowering independently of photoperiod. Putative additional epistatic interactions of HvVRN-H2 with HvGI and HvCO1 point toward a role of HvVRN-H2 as an integrator of photoperiod and vernalization signals (Maurer et al., 2015).

##### Genetic regulation of autonomous and gibberellin response pathways

In addition to the photoperiod and vernalization pathway genes, flowering time in barley is controlled by the *EARLY MATURITY* (*EAM*) loci referred to as *EARLINESS PER SE* (*EPS*) in wheat. The red/far-red light photoreceptor *HvPHYC*, an ortholog of *Arabidopsis PHYC*, was identified to underlie the *EARLY MATURITY 5* (*EAM5)* QTL (Pankin et al., 2014). *HvPHYC* was found to promote light signal transmission to the circadian clock, modulating photoperiodic regulation of floral transition. It was also shown to interact with the *PPD-H1* pathway and to increase *HvFT1* expression not associated with the circadian clock (Nishida et al., 2013; Pankin et al., 2014). Similar mechanisms were discovered in wheat, where *TaPHYC* was found to activate *TaPPD1* and *TaVRN3* in inductive LDs (Chen et al., 2014).

The circadian clock gene *HvLUX1*, an ortholog of the *Arabidopsis* circadian gene *LUX ARRHYTHMO*, was detected as a candidate underlying the barley *EAM10* locus (Campoli et al., 2013). As shown for barley plants carrying a mutation in *EAM5* (*HvPHYC)*, mutations in *EAM10* (*HvLUX1*) altered the expression of *HvPPD-H1* (as well as *CCA1* in barley) and accelerated flowering under both LDs and SDs independently of the circadian clock. Furthermore, as shown for barley *EAM8* mutants (Faure et al., 2012), early flowering of *EAM10* mutants was linked with an up-regulation of *HvFT1* transcription under SDs. In both cases this led to an induction of the LD photoperiod pathway under non-inductive (SD) conditions, suggesting that *EAM10* acts as a repressor of *HvPPD-H1*.

In barley, *HvCEN*, a homolog of *Antirrhinum majus CENTRORADIALIS*, was identified at the *EARLINESS PER SE 2* (*EPS2*) locus on chromosome 2H (Comadran et al., 2012). *Antirrhinum* cen mutants produce terminal flowers instead of indeterminate inflorescences common for wild-type plants without, affecting flowering time (Bradley et al., 1996). Analysis of the *HvCEN* alleles of a diverse set of spring and winter barley cultivars revealed that *HvCEN* facilitated the geographic range extension as well as the gradual separation between spring and winter cultivars (Comadran et al., 2012). A collection of flowering mutants was used to confirm sequence variations within *HvCEN* to be responsible for the observed variation in flowering time. While *CEN* homologs have been identified and characterized in rice (Nakagawa et al., 2002) and maize (Danilevskaya et al., 2008a), none have been identified to date in hexaploid wheat.

The circadian clock gene *HvELF3*, an ortholog of *Arabidopsis ELF3*, was identified to underlie the *EARLY MATURITY 8* (*EAM8*) QTL (Faure et al., 2012; Zakhrabekova et al., 2012). Mutations in *EAM8*, also known as *praematurum.a-8* (*mat-a-8)*, were used since 1961 to facilitate short growing season adaptation and expansion of the geographic range in commercial barley varieties (Zakhrabekova et al., 2012). A loss-of-function mutation of *HvELF3* led to an increased expression of *HvFT1* compared with wild-type plants, causing a day-neutral early flowering phenotype (Faure et al., 2012). Boden et al. (2014) investigated barley plants carrying mutations in the *HvELF3* gene and characterized them as early flowering under non-inductive SD photoperiods. The enhanced expression of *HvGA20OX2* increased GA biosynthesis and expression of *HvFT1.* In spring barley, *HvELF3* is necessary to maintain photoperiodic sensitivity through repression of *HvFT1* and production of active GA via *HvGA20OX2*, a link not previously shown for *AtELF3.*

The phytohormone GA promotes flowering in barley and is essential for normal flowering of spring barley under inductive photoperiods (Boden et al., 2014). One of the gibberellin 20-oxidase genes, *HvGA20OX2*, was identified as the candidate gene underlying the allelic dwarfing gene *sdw1/denso. sdw1/denso* is an important locus selected for in barley breeding programs as the resulting semi-dwarf phenotype has reduced plant height and improved lodging resistance associated with higher yields and quality traits. The *sdw1* mutant was distinguished from the *denso* mutants based on the more strongly reduced expression of *HvGA20OX2* which was also linked to reduced plant height, enhanced grain yield, and lower grain quality (Jia et al., 2011).

### Regulation of Flowering Time in Short-Day Plants Rice and Maize

Rice and maize are tropical SD plants and, unlike wheat and barley, do not require vernalization. However, a high level of conservation of photoperiod pathway genes with more distantly related plants like *Arabidopsis* and temperate cereals is found in both rice and maize. If similar genes controlling flowering are involved in plants that flower under LD photoperiods (such as temperate cereals and *Arabidopsis*), which regulatory mechanisms generate the reverse response to photoperiod in rice?

#### Flowering Time Regulation in Rice

##### Genetic regulation of photoperiod response

Two independent pathways act in response to different photoperiods to control flowering time in rice. Day-light periods of less than 13.5 h accelerate flowering of rice (Itoh et al., 2010). Under inductive SD conditions, a *HEADING DATE 1* (*HD1*)-dependent pathway is activated in which the *AtCO* homolog *HD1* accelerates flowering via activation of the *AtFT* ortholog *HEADING DATE 3A* (*HD3A*) (Kojima et al., 2002). This pathway is conserved between *Arabidopsis* and rice (**Table 3**). For heading initiation under non-inductive LD conditions, an *HD1*-independent pathway is activated (Yano et al., 2000). The activity of genes unique to rice play a major role in conferring the different seasonal flowering responses when compared to LD plants such as *Arabidopsis* and are highlighted in the following sections.

**Table 3:** Rice functional orthologs or homologs of *Arabidopsis thaliana* genes.

Rice	*Arabidopsis*	Function	Chromosome	Reference
**Functional orthologs**				
*OsELF3*	*AtELF3*	Floral promoter	6	Zhao et al., 2012
*OsGI*	*AtGI*	Floral promoter	1	Hayama et al., 2002, 2003
*HD3A*	*AtFT*	Floral promoter	6	Tamaki et al., 2007
*OsMADS14*	*AtAP1*	Meristem identity	3	Komiya and Shimamoto, 2008
*OsMADS50*	*AtSOC1*	Floral promoter	3	Ryu et al., 2009
*OsPRR37*	*AtPRR3/7*	Floral repressor	7	Murakami et al., 2007
*OsRFT1*	*AtFT*	Floral promoter	6	Komiya et al., 2009
**Homologs with pleiotropic or divergent function**				
*HD1*	*AtCO*	Floral repressor in LDs, floral promoter in SDs, affects several agronomic traits	6	Yano et al., 2000; Zhang et al., 2012; Nemoto et al., 2016
**Unique genes**				
*EHD1*	–	Floral promoter in SDs	10	Xue et al., 2008
*GHD7*	–	Floral repressor in LDs	7	Xue et al., 2008
*GHD7.1*	*AtPRR7*	Floral repressor in LDs, floral promoter in SDs, affects several agronomic traits	7	Yan et al., 2013
*GHD8*	*AtHAP3b*	Floral repressor in LDs, floral promoter in SDs, affects several agronomic traits	8	Yan et al., 2011
*OsID1*	–	Floral promoter in SDs	10	Matsubara et al., 2008
*OsMADS51*	–	Floral promoter in SDs	1	Kim et al., 2007


*Functional orthologs have conserved function, homologs have a pleiotropic or divergent function, and unique genes have currently no known functional orthologs nor homologs in *Arabidopsis*. Gene name abbreviations are explained in **Supplementary Table 1**.*

###### HD1-independent pathway under a non-inductive (LD) photoperiod

**FIGURE 3:**
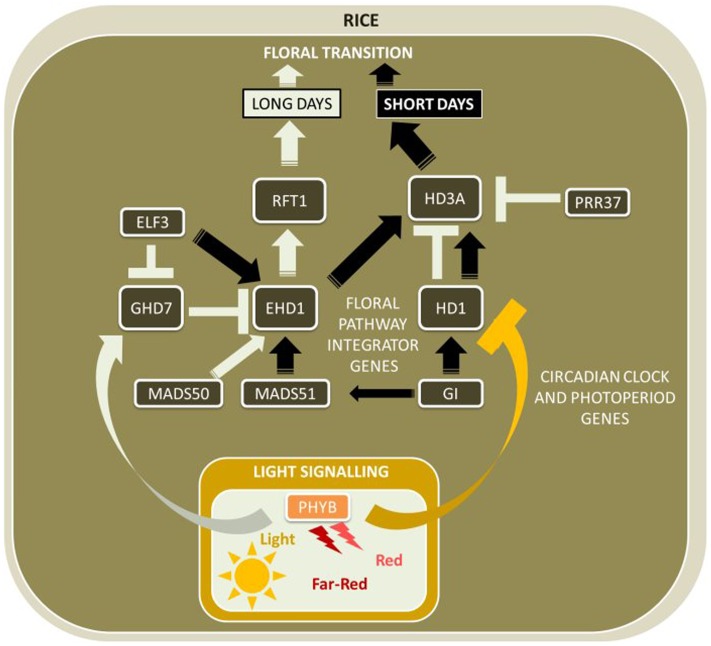
**Major flowering pathway genes of rice (*Oryza sativa*).** Positive and negative regulatory connections are indicated by arrows and lines with T-ends, respectively. White and black arrows or T-ends indicate regulatory connections occurring primarily under long days and short days, respectively. Gene name abbreviations are explained in **Supplementary Table 1**.

As rice does not require vernalization, the photoperiodic pathway is of great significance to control flowering, often referred to as heading date. Similarly as for *Arabidopsis*, the mobile flowering signal *HD3A* is expressed in leaves and the protein is translocated to the SAM where it accelerates floral development (Kojima et al., 2002). The key difference between the genetic regulation of *Arabidopsis* and rice flowering time is that unlike its ortholog *AtFT*, *HD3A* is only expressed under SD conditions (**Figure 3**).

*HD1* is expressed with a diurnal rhythm similar to *AtCO*, with a peak in expression in the afternoon in LD photoperiods (Yano et al., 2000). However, unlike *AtCO*, which activates *AtFT* in LDs, high *HD1* activity in LDs is associated with low expression of *HD3A* (Hayama et al., 2003). *OsGI* is also expressed with a diurnal rhythm similar to its ortholog *AtGI*, and likely has a similar role in regulating *HD1* expression (Hayama et al., 2002, 2003).

Other components of the circadian clock modify the activity of genes acting in the photoperiod pathway in rice. For example, the transcript of the *VRN2*-like CCT-motif gene *GRAIN NUMBER, PLANT HEIGHT, AND HEADING DATE 7* (*GHD7*) is activated by red light signaling via phytochromes in the morning of LDs (Xue et al., 2008). GHD7 represses the expression peak of the B-type response regulator *EARLY HEADING DATE 1* (*EHD1*), a gene without a known homolog in *Arabidopsis*, at the start of the light period to delay flowering under LD. *EHD1* is known to confer promotion of flowering under SDs (the exact mechanism is currently unknown), and mutations in the DNA-binding domain or gene suppression via RNAi decreased *HD3A* expression under SD photoperiods (Doi et al., 2004; Kim et al., 2007). Another gene with a CCT motif, *OsPRR37*, is an ortholog to the circadian clock gene *AtPRR3/7*, and although expressed independently of photoperiod, is only functional to repress *HD3A* under LDs (Murakami et al., 2007). GHD7 was also found to suppress the expression of *HD3A* and to delay flowering in LDs but does not affect *HD3A* expression under SDs, indicating that *GHD7* is further upstream of both *HD3A* and *EHD1* in the gene network that controls rice flowering.

Cultivated rice is widely grown throughout Asia, including geographical areas in northern Asia with long light periods of up to 14.5 h per day during the growth period (Izawa, 2007). To avoid cold temperatures during grain filling common to these regions, photoperiod insensitive, early flowering and rice cultivars are required (Izawa, 2007). Although flowering in rice is normally restricted to SDs, rice cultivars were developed with the ability to flower under LD conditions. The pathway involved in floral activation under LD conditions involves OsMADS50, a homolog of AtSOC1, which activates *EHD1* expression, which in turn activates expression of *RICE FLOWERING LOCUS T 1 (OsRFT1*), the closest homolog of *HD3A* (Komiya et al., 2009; Ryu et al., 2009). *HD3A* and *OsRFT1* are both mobile flowering signals, located very closely on the same chromosome, but with inverse functions - *HD3A* is activated under LD, whereas *OsRFT1* expression is activated under SD conditions. Also, natural variation of *OsPRR37* (Koo et al., 2013) and major-effect HD-QTLs (*HD1*, *HD2*, *HD4*, and *HD5*) (Li X. et al., 2015), were found to modulate photoperiod sensitivity and to adapt rice varieties to cultivation at a wide range of latitudes. To date, no homologs in *Arabidopsis* or temperate cereals are known for any genes of this alternate pathway.

###### HD1-dependent pathway under an inductive (SD) photoperiod

Inductive SD conditions activate a conserved pathway between rice and *Arabidopsis*, in which *HD1* promotes flowering via activation of *HD3A* (Kojima et al., 2002). Therefore, *HD1* has two opposing functions in the flowering network – to promote flowering under SD, but also to inhibit flowering under LD photoperiods via interaction with *HD3A*. Similarly, *OsELF3*, orthologous to *AtELF3*, has a dual role, as it promotes flowering in SDs via activation of *EHD1*, but also represses *GHD7* to promote flowering in LDs (Zhao et al., 2012). Very recently, a novel flowering time pathway unique to monocot plants was discovered with *HD1*, *GHD7*, and *EHD1* as its key genes: HD1 was shown to directly interact with GHD7 to form a complex that specifically bind to a cis-regulatory region in *EHD1* resulting in repression of *EHD1* gene expression only under non-inductive LD conditions (Nemoto et al., 2016).

As a gene unique to rice, *EHD1* is a key enabler of flowering transition under SD conditions by enhancing expression of *HD3A* and *OsRFT1* independently of *HD1* (Doi et al., 2004). *EHD1* expression is activated by blue light in the morning and controlled via *OsGI*.*EHD1* was found to be up-regulated by the MADS-box gene *OsMADS51* (Kim et al., 2007), which itself is also positively regulated by *OsGI* (Kim et al., 2007). The gene *INDETERMINATE1* (*OsID1*) triggers the expression of *HD1* and *HD3A* for flowering under SDs (Matsubara et al., 2008), and as for *OsMADS51*, no known homologs in *Arabidopsis* were detected.

The heterotrimeric heme activator protein (HAP) complex regulates flowering in *Arabidopsis* through binding to the CCAAT box, a *cis*-acting element (Ben-Naim et al., 2006). The gene *GRAIN NUMBER, PLANT HEIGHT, AND HEADING DATE 8* (*GHD8*) encodes a CCAAT box-binding protein belonging to the HAP3 subfamily in rice, and was recently cloned (Yan et al., 2011). *GHD8 is* homologous to *AtHAP3b* and acts upstream of *EHD1*, *HD3A*, and *OsRFT1* in the rice flowering pathway. Under SD conditions, *GHD8* enhances, and under LD conditions inhibits gene expression of *EHD1*, *HD3A*, and *OsRFT1* via a coordinated interaction with *GHD7*.1, a *PSEUDO-RESPONSE REGULATOR* (*PRR*) gene (Yan et al., 2013). *GHD7*, *GHD8*, and *GHD7.1* have pleiotropic effects on heading date, grain yield and plant height, and together with *HD1* are the key rice genes that defines grain yield and environmental adaptability to rice growing regions.

##### Genetic regulation of gibberellin response pathways

GA signaling affects floral organ development and pathways are largely conserved between rice and *Arabidopsis*. The casein kinase I (CKI) gene *EARLY FLOWERING1* (*OsEL1*) is associated with the negative regulation of GA-responsive signaling via post-translational modification of SLENDER RICE 1 (OsSLR1) protein. Mutations in *OsEL1* lead to an enhanced response to GA signaling, and as a consequence to early flowering, lower spikelet fertility and thus lower grain yield under non-inductive LDs. However, a few extremely early flowering rice cultivars contain the mutation in *OsEL1* but can maintain normal GA signaling through a currently unknown mechanism.

The rice gene *SEMI-DWARF1* (*OsSD1*) contributed to the major increases in rice yield during the “Green Revolution” and is involved in GA signaling and biosynthesis (Peng et al., 1999). A similar reduced-height phenotype is the result of a loss-of-function mutation in *OsGA20OX2*, orthologous to the GA biosynthetic gene *HvGA20OX2* in barley (Spielmeyer et al., 2002). *HvGA20OX2* was proposed as the candidate gene underlying *sdw1/denso* locus and confers a reduced plant height phenotype in barley (Jia et al., 2011).

#### Flowering Time Regulation in Maize

##### Genetic regulation of photoperiod response

Maize was domesticated about 9,000 years ago from the wild grass teosinte (*Zea mays* ssp. *parviglumis*), a native to tropical Central America (Matsuoka et al., 2002). Teosinte requires SD photoperiods to flower, and domesticated maize has been adapted since then to cooler temperate regions of North America and Europe (Chardon et al., 2004). Maize post-domestication breeding was particularly driven by selection for genes and loci to adapt flowering time to new growth environments (Mascheretti et al., 2015). Photoperiod insensitive (day-neutral) maize varieties are cultivated in temperate climates, whereas photoperiod sensitive maize varieties are grown in tropical regions.

Flowering time in maize can range from only 35 up to 120 days, highlighting the high level of genetic diversity in maize phenology genes (Colasanti and Muszynski, 2009). Only a few mutations in flowering time genes have been identified so far, which contributes to the lack of current understanding of genetic and regulatory factors that determine maize phenology. Our current knowledge about maize flowering time is mainly based on results from QTL meta-analysis, (transposon) mutagenesis, and comparative genomics studies with the *Arabidopsis* and rice genomes (Buckler et al., 2009; Wei et al., 2015). Key phenology genes are listed in **Table 4**.

**Table 4:** Maize functional orthologs of *Arabidopsis thaliana* genes.

Maize gene	*Arabidopsis* gene	Function	Chromosome	Reference
**Functional orthologs**				
*ZmD8*	*AtGAI*	Gibberellin metabolism	1L	Thornsberry et al., 2001
*ZmDLF1*	*AtFD*	Floral activator	7	Danilevskaya et al., 2008a
*ZmMADS1*	*AtSOC1*	Floral promoter	9	Alter et al., 2016
*ZmPHYA, ZmPHYB, ZmPHYC*	*AtPHYA, AtPHYB, AtPHYC*	Light signaling, photoperiodic regulation	1, 9, 5	Sheehan et al., 2004
*ZmZCN8*	*AtFT*	Floral promoter	8	Lazakis et al., 2011; Meng et al., 2011
*ZmZCN6*	*AtTFL1*	Floral promoter	4	Danilevskaya et al., 2011
*ZmZFL2*	*AtLFY*	Meristem identity	10	Bomblies et al., 2003
**Unique genes**				
*ZmID1*	–	Floral promoter, autonomous pathway	1	Colasanti et al., 2006


*Functional orthologs have a conserved function, and unique genes have currently no known functional orthologs nor homologs in *Arabidopsis*. Gene name abbreviations are explained in **Supplementary Table 1**.*

**FIGURE 4:**
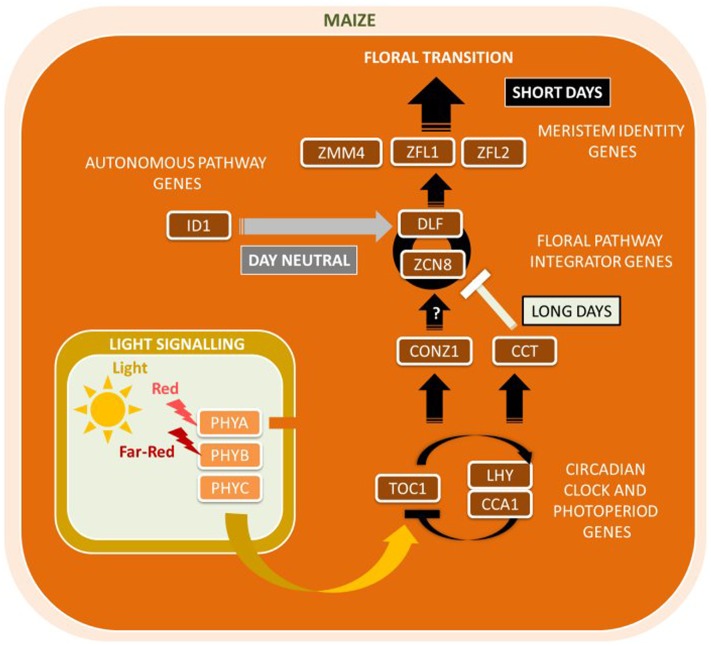
**Major flowering pathway genes of maize (*Zea mays*).** Positive and negative regulatory connections are indicated by arrows and lines with T-ends, respectively. White, black, and gray arrows or T-ends indicate regulatory connections occurring primarily under long days, short days, or independently of day length, respectively. Gene name abbreviations are explained in **Supplementary Table 1**.

Maize contains a large family of *Zea CENTRORADIALIS* (*ZCN*) genes and related to both *AtFT* and *AtTFL* (Danilevskaya et al., 2008a). The most likely candidate for the *FT* ortholog in maize is *Zea CENTRORADIALIS8* (*ZmZCN8*), a gene encoding a PEBP with high sequence similarity to the *Arabidopsis* flowering time gene *AtFT1*. In addition, a second maize florigen, the *ZmZCN8* paralog *ZmZCN7*, was proposed (Mascheretti et al., 2015). Displaying florigen-like characteristics, *ZmZCN8* is expressed only in the leaf phloem of mature leaves and interacts with the FD-like bZIP protein DELAYED FLOWERING1 (ZmDLF1) in the SAM (**Figure 4**). *ZmZCN8* expression is elevated following a diurnal cycle under SDs in photoperiod-sensitive tropical maize, whereas *ZmZCN8* levels remained unchanged in day-neutral temperate maize (Muszynski et al., 2006; Meng et al., 2011). Ectopic expression of *ZmDLF1* resulted in an early flowering phenotype whereas silencing led to a severe delay flowering, as found for its functional equivalent *OsID1* in rice (Matsubara et al., 2008). A putative *AtCO* ortholog in maize, *ZmCONZ1*, was located at a chromosome region syntenic with the rice ortholog *HD1* and exhibits a diurnal expression pattern dependent on photoperiod (Miller et al., 2008). However, and there is currently no evidence that *ZmCONZ1* can directly activate *ZmZCN8* expression in SDs.

Other genes in the maize flowering time network include *Zea FLORICAULA*/*LEAFY* genes (*ZmZFL1* and *ZmZFL2*) (Bomblies et al., 2003). *ZmZFL1* and *ZmZFL2 a*re orthologous to the meristem identity genes *Antirrhinum majus FLORICAULA* (*AmFLO*) and *AtLFY* and share conserved roles in controlling inflorescence architecture and patterning (Bomblies et al., 2003). Among other genes with likely conserved functions between maize and *Arabidopsis* are phytochromes *ZmPHYA*, *ZmPHYB*, and *ZmPHYC* (Sheehan et al., 2004). Unique for maize are Miniature Inverted-Repeat Transposable Element (MITE) sequence insertions detected in *ZmPHYB2* that showed that the absence of the MITE correlated with enhanced *ZmPHYB2* mRNA expression compared to *ZmPHYB2* with MITE (Zhao et al., 2014). This evidence points toward a role of MITEs to inhibit *ZmPHYB2* mRNA expression which ultimately leads to delayed flowering. Similarly, a MITE insertion into the VEGETATIVE TO GENERATIVE TRANSITION (VGT1) locus was associated with early flowering (Castelletti et al., 2014). The CCT domain-containing protein *ZmCCT* was found to be associated with photoperiod sensitivity and to regulate maize flowering time under LD conditions (Yang et al., 2013). The insertion of a CACTA-like transposon into its promoter represses *ZmCCT* gene transcription and thus reduced maize photoperiod sensitivity under LDs. Yang et al. (2013) provided evidence that the CACTA-like transposon was originally inserted in a tropical maize plant sensitive to SDs and was then accumulated selectively post-domestication as maize adapted to a range of LD environments.

##### Genetic regulation of autonomous and gibberellin response pathways

Compared to flowering sensitive to vernalization as found in the temperate grasses wheat and barley or photoperiod-induced pathways as found in tropical rice, endogenous cues are more crucial for flowering than environmental signals in day-neutral temperate maize. The floral promoter *INDETERMINATE1* (*ZmID1*) is a transcriptional regulator of the maize autonomous flowering pathway that functions in developing leaves to promote flowering (Wong and Colasanti, 2007). *ZmID1* encodes a monocot-specific C2H2-type zinc finger protein without any known *Arabidopsis* ortholog and is hypothesized to act independently of the photoperiod pathway (Colasanti et al., 2006). Recent reports show that *ZmID1* expression levels are high in developing leaves, and transcript and protein levels remain stable throughout the light cycle (Wong and Colasanti, 2007; Coneva et al., 2007). Mutations in *ZmID1* lead to an extended vegetative phase (Colasanti et al., 2006). Genetic and expression data has shown that *ZmID1* activates *DELAYED FLOWERING1* (*ZmDLF1*) in the SAM, most likely indirectly (Muszynski et al., 2006; Meng et al., 2011), which is necessary for flowering through its interaction with *ZmZCN8*. Subsequent expression of floral identity genes, such as the floral transition MADS box gene *ZmZMM4*, then initiate reproductive development (Danilevskaya et al., 2008b).

Very recently, transcription and chromatin modifications of *ZmZCN8* and its paralog *ZmZCN7*, a potential second maize florigen, were analyzed during floral transition in day-neutral maize and tropical teosinte (Mascheretti et al., 2015). This study led to the proposal of an alternative epigenetic mechanism of *ZmID1*-mediated regulation of *ZmZCN8* expression in which *ZmID1* establishes chromatin modifications in developing leaves of day-neutral maize to enable the expression of florigen genes *ZmZCN8 and ZmZCN7* in the mature leaf later in development (Mascheretti et al., 2015). By contrast, a different set of *ZmZCN8* chromatin modification patterns were detected in teosinte in response to inductive SDs, which highlights both conserved and unique features of epigenetically controlled flowering time mechanisms between autonomous and photoperiod-dependent pathways in maize.

GA accumulation and signaling has a direct positive affect on flowering time in maize (Thornsberry et al., 2001). The maize gene DWARF8 (ZmD8) encodes a DELLA protein orthologous to both Arabidopsis GIBBERELLIN INSENSITIVE (AtGAI) and the wheat REDUCED HEIGHT mutations TaRHT-B1 and TaRHT-D1, which were used to develop the high-yielding semidwarf varieties of the “Green Revolution” (Peng et al., 1999). Polymorphisms in ZmD8 were associated with differences in flowering time (Thornsberry et al., 2001). Very recently, ZEA MAYS GA REGULATORY FACTOR (ZmGRF) was identified as a new member of the bZIP protein transcription factor family in maize (Xu et al., 2015). ZmGRF transgenic Arabidopsis plants had enhanced GA levels indicating that ZmGRF has a function in plant morphology and development, and Arabidopsis has a currently unknown ortholog of ZmGRF.

## Future Climate Change Scenarios: Adaptation to New Growing Environments and Conditions

Global food production is not increasing fast enough to meet the needs of the rapidly growing human population, and at the same time, global warming as a result of climate change threatens the productivity of existing agricultural land (United Nations Food and Agriculture Organization, 2009). In cereal crops, the correct targeting of flowering time to a narrow seasonal window is directly linked with an increase in grain yield. Climate change and global warming strongly alter phenological cycles and decrease yields of cereals and other crops through rising temperatures and elevated atmospheric carbon dioxide (CO_2_) levels, and more frequent extreme weather events such as drought. This poses a significant challenge to crop growers as they have to adjust crop management practices including sowing dates to achieve flowering at the optimal time. Optimal timing of flowering has also implications for a variety of other traits such as plant vigor, water-use efficiency, and yield (Ni et al., 2009; Kenney et al., 2014). Also, susceptibility to abiotic and biotic stresses and fertilizer requirements vary during the course of plant development. A major challenge for crop breeding is how to plan and adapt breeding efforts to future climate change scenarios.

### Impact of Elevated CO_2_ on Plant Phenology

The Intergovernmental Panel on Climate Change Reports (IPCC) predicts that the current CO_2_ concentrations will more than double by 2050, and by 2100 more than triple (IPCC, 2013). Plants play a central role in mitigating the effects of rising atmospheric CO_2_ through photosynthesis which converts solar energy into energy stored in starch and other carbohydrates. Elevated CO_2_ was shown to increase photosynthesis and growth of many plant species (Long et al., 2004; Wang et al., 2015). Changes in phenology and plant size due to high CO_2_ is believed to be an indirect consequence of the effect of CO_2_ on higher photosynthetic rate resulting in enhanced plant growth. These changes can be highly variable depending on other environmental factors, plant species, and length and level of CO_2_ exposure, with some studies showing that elevated CO_2_ accelerates flowering particularly of crop species with a weaker or no effect on many wild plant species (Springer and Ward, 2007). An early study by Reekie et al. (1994) found that elevated CO_2_ enhanced flowering in LD plans, but delayed flowering in all SD plants grown under inductive photoperiods. By contrast, an experiment that exposed a species-rich temperate grassland to both pure-CO_2_ free-air CO_2_ enrichment (FACE) and infrared warming only detected an effect of higher temperature on phenology but no effect of elevated CO_2_ without experimental warming (Hovenden et al., 2008).

In a meta-analysis conducted by Taub et al. (2008) elevated CO_2_ was found to reduce grain protein concentration of wheat, barley, and rice of 10–15% compared to levels detected at ambient CO_2_ levels. A reduction in protein concentration was also detected for other crops including potato and soybean, but the magnitude of the effect varied was dependent on the experimental facilities and procedures. Such changes in grain nutritional composition have important implications for the malting industry for malting barley varieties, as well as the consumers of crop plant material.

Little research has been conducted so far trying to address the molecular pathways and mechanisms that drive high-CO_2_-mediated changes of flowering time. Elevated CO_2_ was shown to alter the accumulation of photosynthates including sugars and starch (Springer and Ward, 2007). Elevated CO_2_ was shown to interact with both sensing and subsequent transduction of light signals in the *Arabidopsis* photoperiod pathway, which varied with the direction and magnitude of photoperiod (Song et al., 2009). The impact of elevated CO_2_ on time of flowering was linked to enhanced plant growth and an increased number of leaves at flowering. Ward et al. (2012) identified a QTL that affects flowering time at elevated CO_2_ levels, also in *Arabidopsis*, with no connection to photoreceptors. Instead, the gene *MOTHER OF FT AND TFL1* (*AtMFT*), a homolog of the flowering inducer *AtFT* and the inflorescence architecture gene *AtTFL1*, was identified as the underlying candidate gene.

Several reports on the impact of elevated CO_2_ on plant phenology often provide ambiguous results or are even contradicting - elevated CO_2_ leads to early flowering in some species, delays flowering in others, or shows no effect. Clearly, more work is needed to elucidate the mechanisms and causes of rising CO_2_ levels on flowering time and quality traits of plants including crops.

### Impact of Increased Temperature on Plant Phenology

Temperature affects plant development rates in many plant species as well as vernalization in winter crops. Plants require a certain number of degrees accumulated per day above a defined temperature threshold called thermal time, also known as growing degree days (GDD) or units (GDU), to reach the next developmental stage (Bonhomme, 2000). Warmer temperatures often increase the rate of development (Craufurd and Wheeler, 2009). It is forecast that rising CO_2_ levels will also invariably drive temperatures higher, and their combined effects were shown to be more severely affecting flowering time than the individual effects (Hovenden et al., 2008; Johnston and Reekie, 2008). In wheat, elevated CO_2_ had a positive effect on root and shoot biomass, which was alleviated when plants were additionally exposed to high temperatures (Benlloch-Gonzalez et al., 2014). Field-based experiments using elevated CO_2_ combined with supplementary heating (temperature FACE, T-FACE) and different sowing date treatments exposed wheat plants to temperatures ranging from below 0°C to above 40°C (White et al., 2011). This resulted in significantly early heading date as a response to increasing temperature.

Current knowledge on molecular pathways and mechanisms impacted by temperature-mediated changes of flowering time is poor. Recent studies suggest a role of MADS-box genes in response to high temperatures in *Arabidopsis*: *AtFLC*, the best studied temperature-responsive MADS-box gene, was shown to prolong the circadian period at higher temperatures as a means to compensate the circadian clock (Edwards et al., 2006).

## The Potential For Crop Improvement Using Phenology-Dependent Traits

Flowering time genes and regulators often have pleiotropic effects on multiple agronomically important traits, including the number and size of seeds, spikelet fertility, growth vigor, and stress tolerance (Xue et al., 2008; Ni et al., 2009; Andres and Coupland, 2012; Kwon et al., 2015). A major driver of crop evolution and adaptation, flowering time genes are key selection factors for crop breeding.

### Crop Growth Simulation Models to Predict Crop Development: Phenology and Plasticity under Climate Change

The optimization of flowering time of cereal crops to target environments under different environmental conditions is key to adapting future crops to changing environments for increased crop productivity, and to meet the goal to increase world food supply by 70% by 2015 (Anonymous, 2009). When extreme weather events coincide with sensitive growth stages in crops, it can have devastating effects on plant development and yield. Crop models have been developed with the goal to simulate interactions between genotype × environment × cropping system to predict and optimize phenology, crop yields, and other agronomic parameters in response to climate variations and to support ideotype plant breeding (Rötter et al., 2015). Phenological stages of crop plant are simulated in response to photoperiod and temperature, which impacts the development rate and vernalization requirement, as well as their interaction (Keating et al., 2003).

Model-based predictions of these interactions can be used for

(i) ideotyping: identifying breeding targets that define crop growth and development in a given environment,
(ii) agronomic diagnosis: characterizing the growth environment, and
(iii) cultivar choice: facilitating cultivar selection for a given environment and cropping system (Jeuffroy et al., 2014).

The most frequently used genotypic characteristics in crop models relate to phenology traits. For example, in a study representing wheat growing regions in Europe with the aim to optimize wheat yields for future climate scenarios, Semenov et al. (2014) listed nine cultivar parameters, with three of them directly related to plant phenology (phyllochron, day length response, and duration of grain filling). Stresses at booting were also found to impact yield more severely than at anthesis.

In environments characterized by a scarcity of water during growth periods, crop phenology can be the main factor that limits yield potential and can explain much of the yield variability. For example, in Australia, which is characterized by warm winters, hot and dry spring-summers, low-fertility soils and highly variable rainfall, increasingly earlier maturity in the original wheat cultivars introduced from Europe have been a key adaptation to increase yields in these predominantly dry rainfed growing environments (Richards et al., 2014). This allowed crops to flower and fill grain sufficiently early to escape terminal drought stress and high temperatures that were favorable for the spread of diseases. By contrast, in temperate and more humid environments as found for example in Western Europe, biotic stresses including crop diseases are the main contributors of yield limitation and are rarely addressed by model simulations (Jeuffroy et al., 2014).

The model performance of individual models was shown to have limited capability to estimate yield across different experiments consistently (Li T. et al., 2015). To improve the quality of the modeling of climate change impacts on agriculture, long-term empirical data sets of high quality for model calibrations and testing need to be made available. The Agricultural Model Intercomparison and Improvement Project (AgMIP) is composed of international teams and linkages of climate, crop, and economic modeling research that work together to improve crop simulation models^1^. The Knowledge Hub FACCE MACSUR is based in Europe and brings together 18 participating European institutions to facilitate the modeling of climate change impacts on European agriculture^2^.

### Genetic Sequence-Based Selection and Targeted Manipulation of Phenology Genes to Predict Phenology

The potential for crop improvement using phenology-related traits has become a major research focus in recent years (Turner et al., 2005; Cockram et al., 2007; Greenup et al., 2009; Bendix et al., 2015; Müller et al., 2016). A clear understanding of the natural variation at the loci and underlying genes is a key prerequisite to enable the development of varieties adapted to future climates. Phenology gene and allelic information have demonstrated value for breeding improved cereal crops and were used to fine-tune adaptation to different geographic regions and climatic conditions (Porker et al., 2015). This has allowed growers and breeders in the past to produce elite varieties with optimal flowering time for various target environments that achieved high yields at least for current climates (**Table 5**). For example, polymorphisms in vernalization requirement (*VRN*) genes, photoperiod sensitivity (*PPD*) genes, *EAM* loci, as well as *EPS* loci were selected in wheat and barley which together account for much of the genetic variation in flowering time in these crops (Zakhrabekova et al., 2012).

**Table 5:** Targets for QTL or genetic sequence-based selection.

Prediction based on genes or QTL	Target trait	Crop	Reference
QTL	Heading date	Wheat	Bogard et al., 2014
*TaVRN1, TaVRN2, TaVRN3, TaVRN4*	Flowering	Wheat	Brown et al., 2013; Zhang et al., 2015
*TaPPD-D1, TaVRN1*	Flowering	Wheat	White et al., 2008
QTL	Flowering	Barley	Yin et al., 2005
QTL	Flowering	Rice	Nakagawa et al., 2005
*HD1, EHD1*	Flowering	Rice	Wei et al., 2016
QTL	Leaf elongation rate	Maize	Reymond et al., 2003


*Gene name abbreviations are explained in **Supplementary Table 1**.*

Although considerable progress regarding model improvement has been made, difficulties remain that are often linked to high genotype × environment interactions. Gene-based models are a new concept as part of the future direction of crop modeling that utilizes a large amount of genetic data generated by molecular genetics techniques in the laboratory. They have the potential to allow *in silico* identification of the best allelic combination for a given set of environments. For example, Zheng et al. (2013) recently presented a gene-based model to predict wheat heading time across different environments along the Australian wheat belt. The effects of *TaPPD-D1* genes were combined with three homoeologous *TaVRN1* genes (*TaVRN1-A1*, *TaVRN1-B1* and *TaVRN1-D1*) to explain variation for heading time. In this study, the winter allele of *TaVRN-A1* had the strongest effect on delaying heading time compared with the effects of the winter alleles of *TaVRN-B1* and *TaVRN-D1.* The gene-based model was built to predict wheat phenology based on the modeling framework Agricultural Production Systems Simulator (APSIM) using gene parameters for *TaVRN1* and *TaPPD-D1*, showing an improved performance over existing gene- and QTL-based models.

Simulations of crop yield using their allelic values at QTL as an input in multi-environment crop models are promising to enable predictions for the beneficial or adverse effect of a given combination of alleles on plant performance and yield (Tardieu and Tuberosa, 2010). Bogard et al. (2014) proposed a QTL-based model to predict heading time in wheat, also using genotype vernalization requirements and photoperiod sensitivity as model parameters. The main difference to other gene-based approaches is that this model did not make *a priori* assumptions about which exact genes determine model parameters. Earlier examples of gene and QTL-based model prediction of flowering also exist for maize (Reymond et al., 2003), rice (Nakagawa et al., 2005), and barley (Yin et al., 2005).

## Prospects and Challenges

The degree of conservation and functional diversification of phenology genes between *Arabidopsis thaliana* and cereal crops has been researched extensively since the release of cereal crops reference genomes. As a consequence, major discoveries have been made in recent years for wheat, barley, rice, and maize, but further developments are needed to translate these findings into improved yield in the field, particularly in the following areas:

(i) Identification of all genomic regions containing phenology and related genes for agronomically important cereal crops: The next phase of model development and validation is to incorporate genome-wide association mapping (GWAS) and genotyping-by-sequencing (GBS) technologies to maximize the rate of trait discovery and improve phenotypic prediction under diverse environments.
(ii) More extensive genetic characterization of cereal crop germplasms to determine allelic diversity of phenology and related genes and their effects on flowering time.
(iii) Development of diagnostic markers to capture the range of allelic variation for major phenology genes in cereal crops: The utilization of existing phenology gene alleles to combine traditional breeding techniques with modern biotechnology using marker-assisted selection will further increase the efficiency of introgression of favorable alleles into elite crop cultivars.
(iv) Improvements in accurate field phenotyping of phenology-related and agronomic traits across a range of latitudes: Crop responses to a combination of elevated CO_2_ and high temperature can vary depending on the experimental setup, and conclusions are often based on experiments conducted in controlled environment chambers. The development of field facilities to test several environmental factors on field crops remains a major challenge, and comparably little information is currently available on crops responses to elevated CO_2_ × high-temperature interactions under field conditions.
(v) Advancements in simulation methodologies to link gene sequence information and environmental parameters with performance in the field: Crop simulation models have been developed to provide new insights into how cereal crop varieties may respond under different climate change scenarios and associated stresses, and to design model-aided ideotypes tailored to specific cropping environments. A better understanding of the interactions between photoperiod and high temperature is required to predict responses to of future climates. Also, the significant impact of phenology-related genes on grain yield remains to be integrated into such models.

## Author Contributions

CH: reviewed the literature and wrote the paper; CL: developed the project idea and finalized the paper.

## Conflict of Interest Statement

The authors declare that the research was conducted in the absence of any commercial or financial relationships that could be construed as a potential conflict of interest.The reviewer YX and handling Editor declared their shared affiliation, and the handling Editor states that the process nevertheless met the standards of a fair and objective review.

## Footnotes

1 http://www.agmip.org

2 http://www.macsur.eu
