# Supplementary material for: Genetic Architecture of Flowering Phenology in Cereals and Opportunities for Crop Improvement
Source: Front Plant Sci. 2016 Dec 19;7:1906. doi: 10.3389/fpls.2016.01906 (PMC5165254; doi:10.3389/fpls.2016.01906)
Supplement: Supplementary file 1 [file Table_1.DOCX]

Supplementary Material

**Genetic architecture of flowering phenology in cereals and opportunities for crop improvement**

**Camilla Beate Hill^1^*, Chengdao Li^1,2^***

^1^Western Barley Genetics Alliance, Western Australian State Agricultural Biotechnology Centre, School of Veterinary and Life Sciences, Murdoch University, WA, Australia.

^2^Department of Agriculture and Food Western Australia, South Perth, WA, Australia.

*** Correspondence:** Chengdao Li (c.li@murdoch.edu.au), and Camilla Beate Hill (camilla.hill@murdoch.edu.au), Western Barley Genetics Alliance, Western Australian State Agricultural Biotechnology Centre, School of Veterinary and Life Sciences, Murdoch University, 90 South Street, WA 6150, Australia.

**
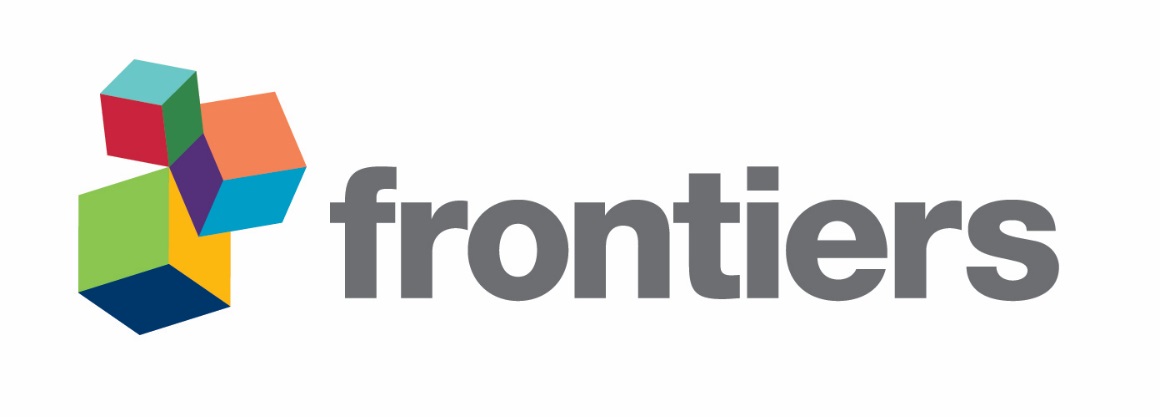
**

**Supplementary Table 1:** Gene names used in Tables 1-5 and Figures 1-4 in alphabetical order with references.

| **Abbreviation** | **Full gene name** | **Reference** |
| --- | --- | --- |
| ***AGL*** | *AGAMOUS-LIKE* | Fujita et l., 2003 |
| ***AP*** | *APETALA* | Yan et al., 2003 |
| ***CAL*** | *CAULIFLOWER* | Yan et al., 2003 |
| ***CCA*** | *CIRCADIAN CLOCK ASSOCIATED* | Wang et al., 1998 |
| ***CCT*** | *CCT-DOMAIN CONTAINING* | Yang et al., 2013 |
| ***CDF*** | *CYCLING DOF FACTOR* | Fornara et al., 2009 |
| ***CEN*** | *CENTRORADIALIS* | Comadran et al., 2012 |
| ***CO*** | *CONSTANS* | Nemoto et al., 2003 |
| ***COL*** | *CONSTANS-LIKE* | Trevaskis et al., 2006 |
| ***CONZ*** | *ZEA CONSTANS-LIKE* | Miller et al., 2008 |
| ***CRY1*** | *CRYPTOCHROME* | Devlin, 2002 |
| ***D*** | *DELLA* | Thornberry et al., 2001 |
| ***DLF*** | *DELAYED FLOWERING* | Danilevskaya et al., 2008 |
| ***EAM*** | *EARLY MATURITY* | Zakhrabekova et al., 2012 |
| ***EHD*** | *EARLY HEADING DATE* | Xue et al., 2008 |
| ***ELF*** | *EARLY FLOWERING* | Faure et al., 2012 |
| ***EPS*** | *EARLINESS PER SE* | Comadran et al., 2012 |
| ***FD*** | *FLOWERING LOCUS D* | Danilevskaya et al., 2008 |
| ***FKF*** | *FLAVIN-BINDING, KELCH REPEAT, F-BOX* | Imaizumi et al., 2003 |
| ***FLC*** | *FLOWERING LOCUS C* | Song et al., 2013 |
| ***FT*** | *FLOWERING LOCUS T* | Yan et al., 2006 |
| ***FUL*** | *FRUITFULL* | Yan et al., 2003 |
| ***GAI*** | *GA INSENSITIVE* | Peng et al., 1999 |
| ***GHD*** | *GRAIN NUMBER, PLANT HEIGHT, AND HEADING DATE* | Xue et al., 2008; Yan et al., 2011; Yan et al., 2014 |
| ***GI*** | *GIGANTEA* | Zhao et al., 2005 |
| ***HD*** | *HEADING DATE* | Nemoto et al., 2003; Nemoto et al., 2016 |
| ***ID*** | *INDETERMINATE* | Colosanti et al., 2006 |
| ***LFY*** | *LEAFY* | Bomblies et al., 2003 |
| ***LHY*** | *LATE ELONGATED HYPOCOTYL* | Wang et al., 1998 |
| ***LUX*** | *LUX ARRYTHMO* | Campoli et al., 2013 |
| ***PHY*** | *PHYTOCHROME* | Pankin et al., 2014 |
| ***PPD*** | *PHOTOPERIOD* | Beales et al., 2007 |
| ***PRR*** | *PSEUDO RESPONSE REGULATOR* | Turner et al., 2005 |
| ***RFT*** | *RICE FLOWERING LOCUS T* | Komiya et al., 2009 |
| ***SOC*** | *SUPPRESSOR OF OVEREXPRESSION OF CONSTANS* | Lee et al., 2004; Ryu et al., 2009 |
| ***TFL*** | *TERMINAL FLOWER* | Comadran et al., 2012 |
| ***TOC*** | *TIMING OF CAB EXPRESSION* | Zhao et al., 2016 |
| ***VRN*** | *VERNALIZATION* | Yan et al., 2006 |
| ***ZCN*** | *ZEA CENTRORADIALIS* | Meng et al., 2011 |
| ***ZFL*** | *ZEA FLORICAULA* | Bomblies et al., 2003 |
| ***ZMM*** | *ZEA MAYS MADS BOX* | Danilevskaya et al., 2008 |
